# Functional Evolution of the Feeding System in Rodents

**DOI:** 10.1371/journal.pone.0036299

**Published:** 2012-04-27

**Authors:** Philip G. Cox, Emily J. Rayfield, Michael J. Fagan, Anthony Herrel, Todd C. Pataky, Nathan Jeffery

**Affiliations:** 1 Hull York Medical School, University of Hull, Hull, United Kingdom; 2 Department of Earth Sciences, University of Bristol, Bristol, United Kingdom; 3 Department of Engineering, University of Hull, Hull, United Kingdom; 4 Département d'Ecologie et de Gestion de la Biodiversité, Muséum National d'Histoire Naturelle, Paris, France; 5 Department of Bioengineering, Shinshu University, Ueda, Japan; 6 Department of Musculoskeletal Biology, University of Liverpool, Liverpool, United Kingdom; University College London, United Kingdom

## Abstract

The masticatory musculature of rodents has evolved to enable both gnawing at the incisors and chewing at the molars. In particular, the masseter muscle is highly specialised, having extended anteriorly to originate from the rostrum. All living rodents have achieved this masseteric expansion in one of three ways, known as the sciuromorph, hystricomorph and myomorph conditions. Here, we used finite element analysis (FEA) to investigate the biomechanical implications of these three morphologies, in a squirrel, guinea pig and rat. In particular, we wished to determine whether each of the three morphologies is better adapted for either gnawing or chewing. Results show that squirrels are more efficient at muscle-bite force transmission during incisor gnawing than guinea pigs, and that guinea pigs are more efficient at molar chewing than squirrels. This matches the known diet of nuts and seeds that squirrels gnaw, and of grasses that guinea pigs grind down with their molars. Surprisingly, results also indicate that rats are more efficient as well as more versatile feeders than both the squirrel and guinea pig. There seems to be no compromise in biting efficiency to accommodate the wider range of foodstuffs and the more general feeding behaviour adopted by rats. Our results show that the morphology of the skull and masticatory muscles have allowed squirrels to specialise as gnawers and guinea pigs as chewers, but that rats are high-performance generalists, which helps explain their overwhelming success as a group.

## Introduction

The rodents are some of the most highly specialised mammals with regard to their feeding apparatus. The defining characteristic of the order is the grossly enlarged pair of incisors, seen in both the upper and lower jaws, which are open-rooted and continue to grow through life [1]. The construction of the incisors, with enamel on the buccal surface and dentine on the lingual, creates differential attrition of the outer and inner surfaces, and causes the incisors to be self-sharpening. The cheek teeth are largely composed of dentine [1] and are separated from the incisors by a large diastema resulting from the loss of the canines and anterior premolars [2]. Rodents have two feeding modes, gnawing at the incisors and chewing at the molars, but owing to a mismatch between the cranial and mandibular lengths, the incisors and molars cannot be in occlusion at the same time. Thus, the two feeding modes are mutually exclusive, and the mandible must be moved anteriorly and posteriorly with respect to the cranium (propaliny) to accomplish both these tasks [3], [4].

**Figure 1:**
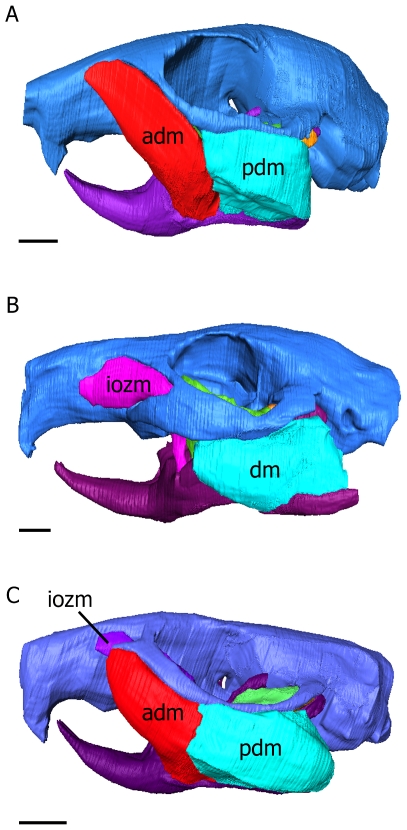
Three-dimensional reconstructions of the skull, mandible, deep masseter and zygomatico-mandibularis of three rodents. (A) sciuromorph (squirrel); (B) hystricomorph (guinea pig); (C) myomorph (rat). adm, anterior deep masseter; iozm, infraorbital part of the zygomatico-mandibularis; pdm, posterior deep masseter. Scale bars = 5 mm.

To cope with the demands imposed by such an unusual dentition and propaliny, the masticatory musculature of rodents has become highly specialised. The masseter is the dominant jaw-closing muscle, forming between 60% and 80% of the masticatory musculature [5], and is divided into three layers in rodents: the superficial masseter, deep masseter and zygomatico-mandibularis (sometimes termed the medial masseter e.g. [6]; see [7], [8] for further detail on nomenclature). In many fossil rodents and also the extant mountain beaver (*Aplodontia rufa*), the masseteric origin is restricted to the zygomatic arch e.g. [9], [10]. This is known as the protrogomorph condition and is thought to be the ancestral morphology [6]. Many other rodents, including all living species except *Aplodontia*, have modified the jaw-closing musculature so that the masseter extends its origin on to the rostrum. This can be done in one of three ways, referred to as sciuromorphy, hystricomorphy and myomorphy [6], [11]. The sciuromorphs, which include squirrels, beavers and pocket gophers, have expanded the deep masseter forwards on to the rostrum to take its origin underneath the widened anterior root of the zygomatic arch (Figure 1A). The hystricomorphs, encompassing the South American rodents plus some Old World forms such as porcupines, jerboas and the springhare, have extended the zygomatico-mandibularis up through the orbit and anteriorly on to the rostrum through the enlarged infraorbital foramen (Figure 1B). Finally, the myomorphs, including mice, rats and their relatives, plus the dormice, have combined the sciuromorph and hystricomorph conditions and expanded both the deep masseter and the zygomatico-mandibularis on to the rostrum, under the zygomatic arch and through the infraorbital foramen respectively (Figure 1C). Further morphological detail of the rodent masticatory muscles can be found in [5], [7].

**Figure 2:**
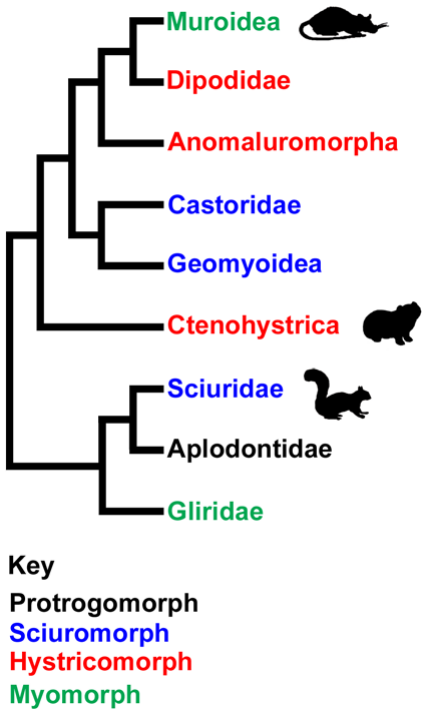
Simplified cladogram of the extant rodents showing the distribution of masticatory muscle morphologies. Topology based on Blanga-Kanfi *et al* [15]. Silhouettes indicate the position of the rat, guinea pig and squirrel within the Muroidea, Ctenohystrica and Sciuridae respectively.

The three morphotypes described above were originally designated as suborders of the Rodentia [11], and this viewpoint persisted for almost a century [12], [13]. However, it has become increasingly clear that the morphology of the masticatory musculature does not neatly fit with the accepted view of rodent phylogeny, and that the sciuromorphs, hystricomorphs and myomorphs do not represent monophyletic groups [6]. Indeed, on examination of some of the more recent molecular phylogenetic work [14]–[16], it can be seen that all three of the muscle morphotypes have evolved more than once within the Rodentia (Figure 2). The underlying reasons for such a high degree of parallelism are still unclear. It seems likely that the physical demands of differing feeding behaviours played a significant role, but as yet we know surprisingly little about the biomechanical implications of the different muscle arrangements, for both incisor gnawing and molar chewing.

Previous biomechanical analyses of rodent mastication have tended to focus on one or two species. Electromyography has been used extensively to study the muscle activation patterns and movements of the lower jaw in rats [17], hamsters [18] and guinea pigs [19]. Quantitative analysis using free body diagrams, based on work by Hiiemae [20] has been used to predict muscle function and to estimate bite forces in a number of myomorphs, such as field mice and voles [21]–[23], the grasshopper mouse [24], the black rat [25] and the Mexican woodrat [26]. Druzinsky also used this method on a larger group of sciuromorph and protrogomorph rodents [27] and concluded that sciuromorphs are more efficient at generating incisor bite force than protrogomorphs. By measuring maximum passive gape and bite force in the deer mouse and grasshopper mouse, it has recently been shown [28] that bite force production is optimised at around 40–50% maximum gape.

This study seeks to investigate the biomechanics of feeding in three rodent species representing the sciuromorph, hystricomorph and myomorph conditions. Specifically, it is hypothesised that the three different arrangements of masticatory muscles will lead to different patterns of stress and strain across the three skull geometries during biting, and that the stress distributions generated by gnawing will be different to those arising from chewing. It is further predicted that differences of muscle and skull morphology between the three rodents will confer benefits or costs to biomechanical performance (e.g. biting efficiency) that reflect an adaptation to a particular habitual or facultative mode of feeding. It has been noted, for instance, that the diet of squirrels contains a high proportion of hard foods, such as seeds and nuts, whereas guinea pigs principally feed on vegetation [1]. Thus, it is hypothesised that the sciuromorph condition is better adapted for gnawing at the incisors, whilst the hystricomorph morphology will produce a more effective grinding action at the molars. It is also hypothesised that myomorphs, whose morphology incorporates elements of both the sciuromorph and hystricomorph conditions, are equally adapted to both feeding modes, but at the cost of biomechanical performance in comparison with the specialist forms.

Biomechanical performance in terms of the stresses and strains generated across the skull by gnawing and chewing will be studied in these rodents using the technique of finite element analysis (FEA). Although originally developed as an engineering tool, FEA has been widely used in recent years to model stress and strain in complex biological objects, frequently vertebrate skulls [29]–[33]. It is of particular use in this type of study as it allows the effect of multiple loading conditions to be investigated in the same skull, without the need for numerous *in vivo* experiments. FEA also enables us to study the effect of non-realistic loading conditions, such as using the muscle proportions of one rodent on the skull of another, which can be used to test whether the muscle morphology is optimised for certain outcomes, such as minimising stress or maximising biting efficiency.

The results of this study will help to further our understanding of how the arrangement of masticatory muscles can affect the biomechanical performance of the skull. If, as hypothesised, it can be shown that the three muscle morphologies are adapted for different feeding strategies, then this may provide an explanation of why each of the three morphotypes appears to have evolved multiple times independently within the Rodentia, and why certain groups within the Rodentia are particularly successful.

## Results

### Stress distribution across the skull

**Figure 3:**
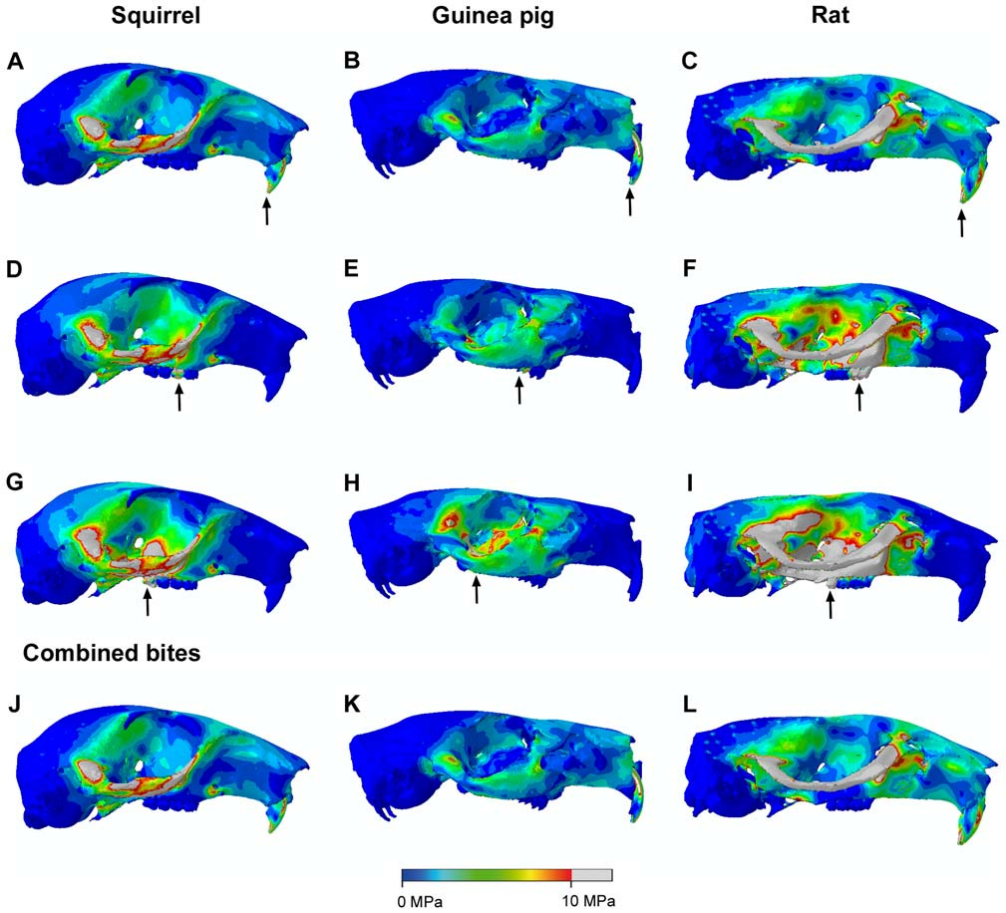
Predicted distribution of von Mises stresses across the skull. Arrows indicate the biting tooth: (A–C) incisor bites; (D–F) unilateral bites on M1; (G–I) unilateral bites on M3; (J–L) maximum von Mises stress experienced by each element across unilateral and bilateral bites on every tooth. (A,D,G,J) squirrel; (B,E,H,K) guinea pig; and (C,F,I,L) rat. Grey areas indicate von Mises stresses exceeding 10 MPa.

**Figure 4:**
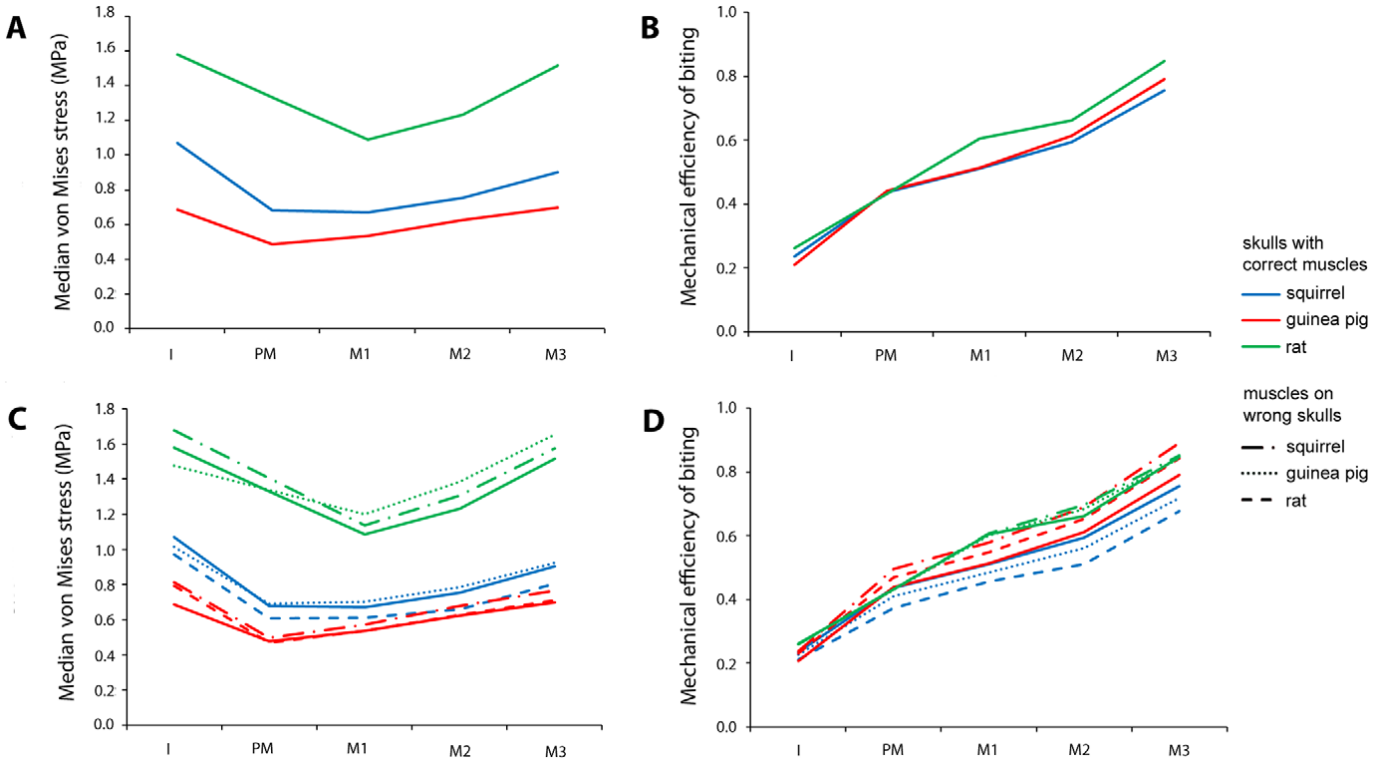
Biting performance of rodents at each tooth. (A) Median von Mises stresses on the skull and (B) mechanical efficiency of biting (predicted bite force divided by total applied muscle force) in squirrels, guinea pigs and rats. (C) Median von Mises stresses and (D) mechanical efficiency of biting of each of the three models with the squirrel, guinea pig and rat muscle configurations applied in turn. I, incisor; PM, premolar (absent in rats); M, Molar.

The von Mises stress patterns resulting from bilateral gnawing at the incisors and unilaterally chewing at the first and last right cheek tooth in the squirrel, guinea pig and rat are shown in Figure 3. The colour scale on each of the skulls in Figure 3 is identical (i.e. 0 to 10 MPa) so that the results can be directly compared. Overall, it can be seen that the rat appears to be experiencing the highest stresses across the skull and the guinea pig the lowest stresses. This is confirmed by the median von Mises stress values illustrated in Figure 4A. In all bites in all rodents, the zygomatic arch is the most highly stressed region of the skull, from the anterior root on the rostrum, all the way along its length to the zygomatic process of the squamosal. Stress patterns along the zygomatic arch are different between the three rodents, but very similar across all bites in each rodent species. The orbital wall generally experiences low stress during incisor biting, but there is a region of higher stress running along the posterior margin of the orbit, between the zygomatic arch and the top of the orbit. The orbital wall, particularly the ventral half, becomes more highly stressed during molar biting, with the stress increasing the closer the bite point is placed to the TMJ. The stress magnitudes are highest in the working side orbit during unilateral biting. High stresses are seen in the rostrum during incisor biting (although not as high as those in the zygomatic arch or orbit), especially along the dorsal and ventral margins of the lateral surface, but, as might be expected, very little stress is experienced in the rostrum during molar biting. The dorsal aspects of the rostrum and cranium show low stresses during incisor biting except for a line running between the anterior roots of the zygomatic arch at the approximate level of the fronto-maxillary suture. During molar biting a small area of stress is seen on the cranium above the posterior half of the working side orbit. The more distal the bite point on the tooth row, the greater in size the area of stress on the cranium. The posterior portion of the skull, particularly the occipital region and auditory bulla, remains largely unstressed in all bites. However, the temporal region of the rat skull, particularly around the origin of the temporalis muscle, does show some stressed areas which are not seen in the squirrel and guinea pig.

The bottom row of images in Figure 3 shows the combined effect of all possible bites in each of the three rodents. The contour maps indicate the maximum von Mises stress experienced by each element over all bites. Although the magnitude of the stresses varies between the rodents (from high in the rat to low in the guinea pig), it is notable that the pattern of stress across the skull is similar in all three models. The highest stresses are seen in the zygomatic arch and orbit, with slightly lower stresses seen on the dorsal and ventral surfaces of the rostrum.

Figure 4A shows the median von Mises stress experienced by the skull elements in the three rodent models during biting at different teeth. This confirms the results obtained from the contour maps that the guinea pig model is experiencing the lowest stress and the rat is experiencing the highest, over all possible bites. The median stress experienced by each model increases as the bite point moves along the molar tooth row towards the TMJ. However, it is notable that the incisor bites do not follow this pattern and, in fact, generate higher stresses than bites on M1. This is probably due to the effect of the rostrum, which is completely unstressed during molar biting, but experiences stress during incisor biting. Therefore, a greater proportion of the elements in each model are experiencing stress during gnawing than during chewing, which reduces the negative skew of the stress distribution, and hence increases the median stress. It is also clear from Figure 4A that this effect is much more pronounced in the squirrel than in the other two rodents. In guinea pigs and rats, the median von Mises stress incurred by incisor biting is respectively 41% and 45% greater than that generated by biting on the first cheek tooth. However, in squirrels, the median stress increases by 57% between premolar and incisor bites, indicating that squirrels are generating much more rostral stress during gnawing than guinea pigs and rats.

### Bite force

Table 1 gives the bite forces predicted by the FE models for biting at each tooth. The incisor bite is a bilateral bite measured at a node on both teeth. The molar bites were unilateral, and the predicted bite forces are the means of the bites on both sides. It can be seen that bite force increases as the bite point moves distally along the tooth row, as would be expected from simple mechanics. In absolute terms, the guinea pig produces the lowest bite force at all teeth, despite being the largest of the three rodents (skull length of 59 mm compared to 55 mm and 43 mm for the squirrel and rat respectively). This is due to the comparatively low masticatory muscle mass of the guinea pig [7]. The squirrel produces the largest bite force of all the three rodents under study at each tooth. The predicted incisor bite forces for guinea pig (18.48 N) and rat (24.65 N) correspond well with our *in vivo* measurements of 19.45±2.53 N for the guinea pig and 31.12±10.75 N for the rat. Furthermore, the value predicted for the rat incisor bite is also very similar to the mean value of 24.3 N measured by Robins [34].

**Table 1:** Average predicted bite force (N) and mechanical efficiency of biting at each tooth.

	Bite Force			Mechanical Efficiency		
	Squirrel	Guinea pig	Rat	Squirrel	Guinea pig	Rat
**I**	26.15	18.48	24.65	0.24	0.21	0.26
**PM**	48.68	38.79	-	0.44	0.44	-
**M1**	56.43	45.32	54.71	0.51	0.51	0.58
**M2**	66.39	54.10	62.13	0.60	0.61	0.66
**M3**	83.63	69.79	76.78	0.76	0.79	0.82

Abbreviations: I, incisor; M1, first molar; M2, second molar; M3, third molar; PM, premolar.

In order to compare simulated bite performance in the three rodents without the confounding variable of size, the predicted bite force was divided by the total applied muscle force to calculate the mechanical efficiency of biting (Table 1). This metric is a measure of how efficiently muscle force is translated into bite force [32]. It can be seen from these results that squirrels are more efficient at biting at the incisors than guinea pigs, but that guinea pigs outperform squirrels at the distal molar teeth (Figure 4B). Even more notable is that rats are more efficient than either of the other two rodents in all incisor and molar bites (premolars are lacking in rat skulls).

### Effect of muscle configuration

The effect of changing the masticatory muscle arrangement on the median von Mises stress across the skull is shown in Figure 4C. It can be seen that stress is minimised by the ‘correct’ muscle configuration in the case of molar bites on the rat skull and most bites on the guinea pig skull (rat muscles on the guinea pig skull produce a very similar median stress at the premolar). However, this is not the case for incisor bites on the rat skull, in which guinea pig muscles lower stress compared to the *in vivo* muscle proportions, or for the squirrel skull, in which the rat muscle arrangement lowers median stress compared to the squirrel muscles across all bites. Indeed, it can be seen that no matter what the skull geometry, the rat muscle configuration will always result in lower stress than the squirrel muscle arrangement.

Figure 4D shows the effect of altering the muscle configuration on another performance metric, the mechanical efficiency of biting, as outlined above. It is clear that changing the muscle proportions on the squirrel skull away from those seen *in vivo* has a negative impact on biting efficiency. Precisely the opposite is true for the guinea pig - applying the rat and squirrel muscle arrangements to the guinea pig skull produces an increase in efficiency in both cases. Changing muscle proportions on the rat model to those of squirrels or guinea pigs has very little effect, with the only noticeable change being a slight increase in the mechanical efficiency of bites on the second molar

**Figure 5:**
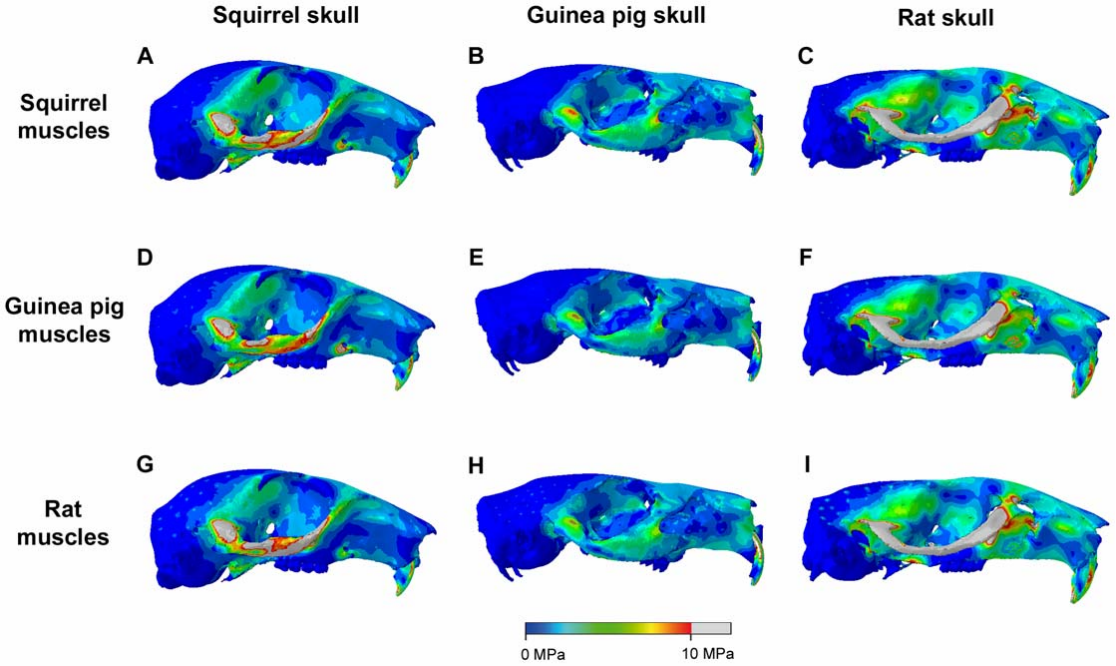
Predicted distribution of von Mises stresses across the skull. Incisor biting in squirrel (A,D,G), guinea pig (B,E,H) and rat (C,F,I), each loaded with squirrel (A–C), guinea pig (D–F) and rat muscles (G–I). Grey areas indicate von Mises stresses exceeding 10 MPa.

**Figure 6:**
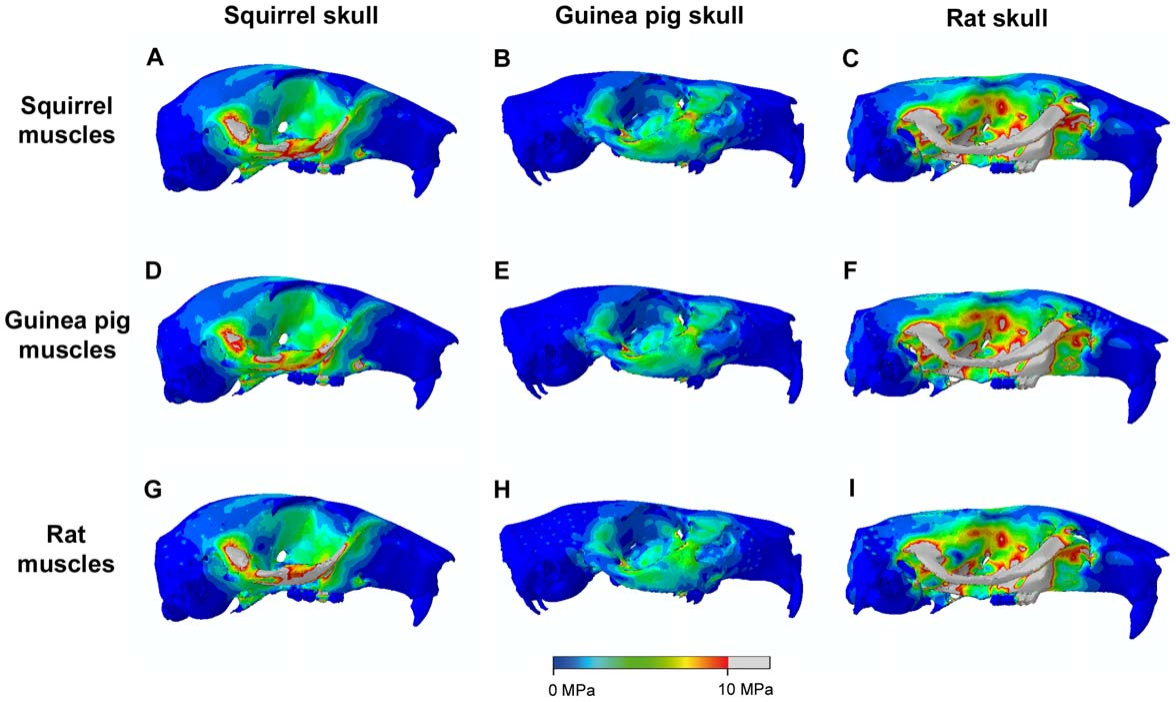
Predicted distribution of von Mises stresses across the skull. Unilateral biting on M1 in squirrel (A,D,G), guinea pig (B,E,H) and rat (C,F,I), each loaded with squirrel (A–C), guinea pig (D–F) and rat muscles (G–I). Grey areas indicate von Mises stresses exceeding 10 MPa.

**Figure 7:**
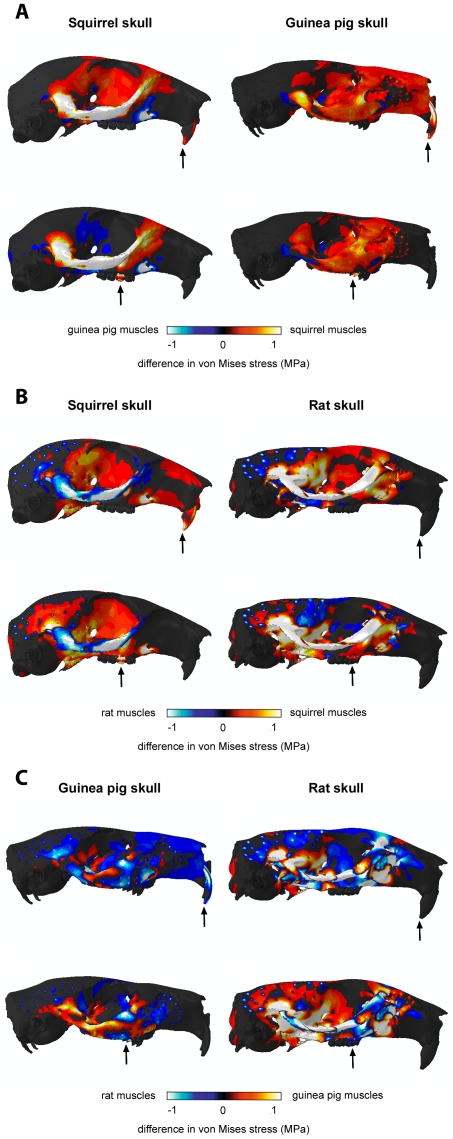
Contour maps showing the difference in von Mises stresses experienced by each model when loaded with different muscle configurations. (A) Stress generated by guinea pig muscles subtracted from stress generated by squirrel muscles. (B) Stress generated by rat muscles subtracted from stress generated by squirrel muscles. (C) Stress generated by rat muscles subtracted from stress generated by guinea pig muscles. Positive results represented by hot colours, negative results by cool colours. Dotted pattern in temporal region of the rat (B and C) indicates the nodes from which the temporalis muscle originates and is a result of the large temporalis of the rat. Arrows indicate the biting tooth.

Figures 5 and 6 show the von Mises stresses generated across the skull by applying the relative muscle proportions of each rodent in turn. Figure 5 shows incisor biting and Figure 6 shows unilateral biting at the right first molar. Although differences are present in the outcomes of these analyses, they are reasonably small and difficult to discern. Hence, to aid understanding of the impact of changing muscle proportions, the results have been represented as contour maps of the difference in von Mises stress experienced at each element. Figure 7A shows the difference between applying squirrel and guinea pig muscle proportions to the squirrel and guinea pig skulls (i.e. Figure 5D subtracted from Figure 5A, Figure 5E subtracted from Figure 5B, and so on). Negative results are represented by cool colours and positive results by hot colours. It can be seen that the squirrel muscles generate higher von Mises stresses across both skulls except around the origin of the superficial masseter on the rostrum. Also, the guinea pig muscles increase stress in the orbital wall of the squirrel skull during molar biting. Figure 7B illustrates the difference between squirrel and rat muscle proportions on the squirrel and rat models. Here it can be noted that squirrel muscles lead to higher stresses in the zygomatic arch of the rat model, whereas rat muscles lead to higher zygomatic stresses in the squirrel model. The orbital wall is generally more highly stressed by squirrel muscles, except in the case of molar biting on the rat model. Rat muscle proportions result in higher stresses in the temporal region in incisor biting, but the squirrel muscles generate greater temporal stresses during molar biting. In all bites, the rat muscles can be seen to be creating localised high stresses around the nodes from which the temporalis muscle originates. This is a consequence of the relatively much larger temporalis of the rat compared to that of the squirrel (Table 2). The same effect is seen between the rat and guinea pig muscle configurations (Figure 7C). The dorsal and ventral surfaces of the squirrel rostrum experience higher stresses during incisor biting when squirrel muscles are applied, but during molar biting and on the rat model, there is little difference in rostral stresses between squirrel and rat muscles. Lastly, the differences between guinea pig and rat muscles proportions applied to the guinea pig and rat models are shown in Figure 7C. It can be seen that the zygomatic arch in these models is a patchwork of areas more highly stressed by guinea pig or by rat muscles. The rostrum of both models tends to experience higher stresses when the rat muscle proportions are applied, whereas the guinea pig muscle proportions tend to generate higher stresses in the posterior orbit and around the orbital foramen, particularly during molar biting.

**Table 2:** Muscle forces (N) applied to each side of each model.

Muscle	Squirrel		Guinea pig		Rat	
	Force	%	Force	%	Force	%
Superficial masseter	10.29	18.6	12.53	28.4	5.95	12.7
Anterior deep masseter	8.79	15.9	5.64	12.8	6.01	12.8
Posterior deep masseter	9.49	17.2			11.49	24.5
Anterior ZM	6.88	12.5	5.51	12.5	1.16	2.5
Posterior ZM	2.02	3.7	1.63	3.7	1.03	2.2
Infraorbital ZM	-	-	3.73	8.4	1.94	4.1
Temporalis	4.19	7.6	4.27	9.7	9.56	20.4
Internal pterygoid	10.26	18.6	8.22	18.6	7.44	15.9
External pterygoid	3.29	6.0	2.60	5.9	2.36	5.0
**Total**	**55.20**	**100**	**44.13**	**100**	**46.94**	**100**

Abbreviations: ZM, zygomatico-mandibularis.

Whilst there is a great deal of information in Figures 5, 6, 7, some overall trends can be drawn from these analyses. Squirrel muscle proportions, when applied to the wrong skulls, tend to increase stress on the rostrum, zygomatic arch and in the anterior part of the orbit. Guinea pig muscle proportions tend to result in lower stresses overall, but if they do generate higher stresses they are generally towards the rear of the orbit. Thus, squirrel muscles appear to be directing stress rostrally, i.e. towards the incisors, whereas the guinea pig muscles tend to direct stress more caudally, towards the molars. Rat muscle proportions on the wrong skull tend to elevate stresses in parts of the zygomatic arch and in the temporal region. This latter effect is largely the result of the increased relative size of the temporalis muscle in the rat.

## Discussion

Finite element models of three rodent species, *Sciurus carolinensis*, *Cavia porcellus* and *Rattus norvegicus*, were successfully constructed, loaded and solved. The bite force values predicted by the models were very close to the values measured by the authors and previous researchers [21] and it was thus concluded that substantial confidence could be placed in the validity of the models.

As hypothesised, incisor gnawing and molar chewing gave rise to very different patterns of stress across the skull. Incisor bites gave rise to large stresses in the rostrum and the posterior orbit, whereas molar bites tended to stress the whole orbital region, with particularly high stresses in the ventral orbit, but not the rostrum (as might be expected from the position of the molar teeth). In all bites, the zygomatic arch experienced the largest stresses, almost certainly as a result of the large muscle mass attaching directly to this part of the skull. In all mammals, a large amount of muscle attaches to this, often slender, rod of bone [5], and rodents are no exception, with both the deep masseter and the zygomatico-mandibularis pulling down on the zygomatic arch [7]. Many biomechanical studies of mammal crania that have simulated feeding with FEA have found that the zygomatic arch is a highly stressed area [32], [33], [35]. In a recent study on primates [36], it has been proposed that a soft tissue structure, the temporal fascia, may be counteracting the large forces pulling down on the zygomatic arch. Despite careful dissection work, we were unable to find a temporal fascia in the rodents, and no such structure is mentioned in previously published accounts of rodent dissection. It should be noted that the models currently assume that all muscles are 100% active during both gnawing and chewing. If this is not the case [20], it may reduce the stress experienced by the zygomatic arch. However, the close correspondence between the predicted and measured bite forces indicates that the models are replicating *in vivo* biting fairly closely.

Despite the substantial differences in skull geometry and muscle morphology, the general pattern of stress across the skull was similar in all three rodents (Figure 3). However, there was large variation in the magnitude of stress experienced by each rodent. Across all bites, the guinea pig experienced the lowest mean stress and the lowest maximum stress of the three rodents, probably due in large part to its low muscle mass relative to skull volume [7]. It can be seen from Figure 3 that the guinea pig zygomatic arch is noticeably less stressed that its counterpart in the squirrel and rat. This is likely the result of many factors: the relatively low overall muscle mass of guinea pigs; the relative dominance of the superficial masseter (which originates from the rostrum) compared to the deep masseter (which attaches directly to the zygomatic arch); and the more robust morphology of the zygomatic arch in this species. The rat shows the greatest stresses across the skull, in all bites, reflecting the large muscle mass compared to skull volume in this species [7]. This is particularly notable in the zygomatic arch and orbital wall, and also in the temporal region where the relatively large temporalis muscle of the rat generates higher stresses than are seen in the squirrel and guinea pig skulls.

The analysis of bite efficiency (Figure 4B) demonstrated that the squirrel is more efficient than the guinea pig at translating muscle force into bite force at the incisors. This supports the hypothesis proposed above that the squirrel morphology is adapted for incisor gnawing, which correlates well with the known diet of this species – squirrels are hard-food specialists, spending a great deal of their time gnawing nuts and seeds [1]. Interestingly, although squirrels demonstrate a very efficient incisor bite, they also show the greatest increase in median stress across the skull between molar biting and the incisor biting (Figure 4A), well over 10% more than that seen in guinea pigs and rats. Therefore, it appears that squirrels are optimised for incisor bite efficiency but not stress minimisation. As has been noted by other researchers, there is no evidence to indicate that mammalian skulls are operating close to the yield stress of bone [32], thus it may be that squirrels are able to incur the risk of higher stresses in order to gain the benefit of increased bite force at the incisors. From the analysis of bite efficiency alone, it is unclear whether the increased gnawing efficiency and increased skull stress seen in squirrels is a product of the skull morphology or muscle arrangement. However, by examining the results of the FE analyses with swapped muscles, it can be seen that all models with squirrel muscle proportions applied have increased stresses in the rostrum and anterior orbit (Figures 7A and 7B), a higher median von Mises stress across the skull (Figure 4C) and increased biting efficiency at the incisors (Figure 4D). Thus it can be inferred that it is the muscles, rather than the skull morphology, that are leading to the higher stresses and gnawing efficiency.

In comparison to squirrels, guinea pigs show an increased efficiency of biting at the molars (Figure 4B), which supports the hypothesis that the morphology of this species is adapted for molar chewing. This conclusion fits well with ecological observations that guinea pigs are mostly grazers that use their cheek teeth to grind down grasses and other vegetation [1]. The results from the analyses with swapped muscle proportions demonstrate that the guinea pig muscles tend to reduce skull stress during gnawing but increase stress during chewing (Figure 4C). Furthermore, the guinea pig muscles on the wrong skulls tend to increase stress towards the back of the skull (Figures 7A and 7C), indicating that here as well it is the muscle arrangement more than the skull morphology that is adapted to molar chewing.

Of the three rodents studied here, the rat has the most efficient bite at all teeth (Figure 4B). Thus, the rat is the most versatile of these species, able to perform well in both gnawing and chewing, unlike the squirrel and guinea pig which have specialised in incisor and molar biting respectively, yet it has not compromised biting efficiency to attain this versatility. The rat also incurs the greatest stresses across the skull (Figures 3 and 4A). However, these stresses are largely a product of the large muscle mass of this species. When the rat muscle proportions are applied to other skulls, it can be seen that they tend to reduce overall stress compared to squirrel and guinea pig muscle arrangements on the same skull (Figure 4C), particularly on the squirrel skull (Figure 7B). Comparing the bite efficiency of different muscle proportions on the rat skull, it can be seen that there is very little difference between the three arrangements (Figure 4D). Thus, it appears that while the rat muscles are adapted to minimise stresses across the skull, the rat skull is adapted to maximise biting efficiency. These two factors have given rats the ability to perform well in all bites, allowing them to become highly successful generalist feeders. This may go a long way to explaining the overwhelming success of both the species *Rattus norvegicus* and the myomorph rodents as a whole, particularly the subfamily Murinae, which contains well over 500 species distributed widely across Europe, Asia, Africa and Australia and contains some of the most detrimental wide-spread invasive species among vertebrates [37], [38].

The models presented in this analysis were necessarily simplified representations of the highly complex interactions of muscle, teeth, bone and food that occur *in vivo*. Previous research has indicated that gape angle can affect maximum bite force [28], [39] and stress patterns across the skull [40]. It has been noted that the proportion of fast- and slow-twitch fibres in the masticatory muscles can vary between taxa and may affect bite force [41]. In addition, rodent molar bites are frequently a great deal more complicated than the static loads simulated here, with wide lateral excursions of the mandible, movement of the mandibular condyles and asymmetric, non-maximal activation of the muscles [18]–[20]. Future models would be improved by taking into account some of these complexities.

The results of this analysis have given insights into how the skull and muscle morphology of the squirrel, guinea pig and rat are adapted to particular dietary niches. Further analysis of other rodent species is needed to understand if biting efficiency at different parts of the tooth row is specific to these three rodent species or an inherent property of the sciuromorph, hystricomorph and myomorph muscle arrangements. If the latter is the case, this may shed a great deal of light on why the three different muscle arrangement evolved from the primitive morphology, and why each has evolved multiple times, independently within the Rodentia.

## Materials and Methods

### Sample

The grey squirrel (*Sciurus carolinensis*), domesticated guinea pig (*Cavia porcellus*) and brown rat (*Rattus norvegicus*) were selected to represent the sciuromorph, hystricomorph and myomorph morphologies respectively. All specimens were from formalin-fixed collections maintained at the University of Liverpool, which had been obtained for earlier studies [7], [40]. Rats were previously supplied post-mortem by Charles River Laboratories International, Inc. (Wilmington, MA, USA), guinea pigs were provided post-mortem by Biomedical Services, University of Liverpool, and squirrels were supplied post-mortem from Lyme Park, Manchester, UK. These species were chosen as they have all been well-studied previously and represent a typical member of each morphotype (i.e. none has unusual specialisations for feeding beyond those seen in all rodents). In order to select an individual close to the centre of the normal range of intraspecific variation, a number of specimens of each species (eight rats, eight guinea pigs and seven squirrels) were imaged using micro-computed tomography (microCT). Imaging was carried out in the Department of Engineering, University of Hull. Field of view (FOV) varied from 27 to 50 mm and slice thickness ranged from 0.047 to 0.076 mm. The total number of slices ranged from 990 to 1160. A geometric morphometric analysis was then performed on a set of anatomical landmarks taken from each stack of microCT images. The results of this analysis allowed the individual with the ‘most average’ morphology to be selected for each species. Full details of this technique are given in [40].

### Model creation

A finite element model was created of each of the three rodent individuals selected by the geometric morphometric analysis. The initial geometry was created from the microCT images using Amira 5.3.2 (Mercury Systems Inc., Chelmsford, MA, USA). The skull, teeth and periodontal ligament were all rendered separately so that they could be assigned separate elastic properties. For the same reasons, the enamel, dentine and pulp layers of the incisors were separated; however, these components were not so easily distinguishable in the molars (due to the small size of the cheek teeth in the squirrel and rat, and the interdigitated nature of the enamel and dentine in the guinea pig). Hence, the molars were modelled as a single volume in all three models. The completed models were converted to three-dimensional meshes in Hypermesh 10.0 (Altair Engineering Inc., Troy, MI, USA). Each model was composed of between 800,000 and 1.2 million tetrahedral linear elements with an average size of 0.25 mm. This is well below the element size of 0.92 mm at which the results of an FEA of a pig skull were found to converge [42].

### Material properties

The six separate volumes in each model (bone, molar teeth, incisor enamel, incisor dentine, incisor pulp cavity and periodontal ligament) were modelled as linearly elastic and were each assigned different values of Young's modulus to reflect the variation in stiffness of each of these tissues. Owing to the small size of the specimens in this analysis, it was assumed that the skulls were composed entirely of cortical bone. This assumption was felt to be justified by recent research on felids [43] that demonstrated negative allometry between cortical bone volume and total skull bone volume (i.e. smaller skulls have a greater proportion of cortical bone). The values assigned to bone, molars, enamel and dentine were the means of measurements taken from dry, sectioned skulls of each species using a nano-hardness tester with a Berkovitch diamond indenter (CSM Instruments S.A., Peseux, Switzerland). This work was carried out at the Department of Engineering, University of Hull. The values for pulp cavity and periodontal ligament were based on previous research [40], [44]. Values for Poisson's ratio for all six materials were taken directly from [44]. Table 3 lists the Young's modulus and Poisson's ratio assigned to each volume.

**Table 3:** Material properties of cranial and dental components.

Component	Squirrel		Guinea pig		Rat	
	*E*	*ν*	*E*	*ν*	*E*	*ν*
Bone	17,850	0.30	18,800	0.30	19,920	0.30
Incisor enamel	80,430	0.33	68,600	0.33	62,370	0.33
Incisor dentine	24,460	0.31	22,620	0.31	23,600	0.31
Incisor pulp cavity*	2	0.45	2	0.45	2	0.45
Molar tooth	30,000	0.30	30,000	0.30	30,000	0.30
PDL*	50	0.40	50	0.40	50	0.40

Abbreviations: *E*, Young's modulus (measured in MPa); *ν*, Poisson's ratio; PDL, periodontal ligament.

* , values taken from literature [29].

### Constraints

Three or four nodes (representing unilateral and bilateral biting) in each model were constrained to prevent translation and rotation in space when the muscle loads were applied. A node on the ventral surface of the zygomatic process of the squamosal bone was constrained on both sides of the model to simulate the temporo-mandibular joint (TMJ). The node on the left TMJ was constrained in all three dimensions, but the node on the right TMJ was only constrained in the antero-posterior and dorso-ventral axes, so as to allow medio-lateral expansion and contraction of the skull. In addition, a node was constrained at the bite point(s) in the axis of the bite. At the molars this was deemed to be perpendicular to the occlusal plane, but at the incisors it was modelled at 75° to the occlusal plane, based on previous sensitivity studies [40]. All incisor bites were assumed to be bilateral because the close apposition of the incisors prevents the possibility of unilateral biting. With regard to molar biting, it was noted that rats habitually chew bilaterally [17], whereas both bilateral and unilateral chewing occur in guinea pigs [19]. No published data could be found regarding chewing in squirrels. Hence, all possible molar bites were modelled: unilateral on each tooth (left and right sides) and bilateral on each pair of molars.

### Muscle loads

The jaw-closing muscles of the squirrel, guinea pig and rat were studied in great detail using both traditional dissection and contrast-enhanced microCT [7], [45] in order to determine accurate muscle origins and orientations. Eight or nine muscles were simulated on each side of each model: the superficial masseter; the anterior and posterior parts of the deep masseter; the anterior, posterior and infraorbital parts of the zygomatico-mandibularis; the temporalis; and the internal and external pterygoids. Reflecting the variation in masticatory muscle morphology, the infraorbital portion of the zygomatico-mandibularis was absent from the squirrel model and the deep masseter was modelled as a single muscle in the guinea pig (see [7] for morphological details). Physiological cross-sectional areas of the masticatory muscles were calculated from muscle volume divided by mean fibre length, measured from the contrast-enhanced microCT scans and subsequent three-dimensional reconstructions of the muscles (such as those shown in Figure 1; see also [7]). Although traditional CT scanning would be unable to provide data on fibre length, it has been shown recently that contrast-enhanced microCT can resolve the detail of muscle fascicles [45]. By dissecting the masticatory musculature of the rat and guinea pig specimens used in the validation study (see below), it was shown that fibre lengths taken from contrast-enhanced microCT scans were within 1.5 mm of *ex vivo* data in most cases. Pennation angle was not taken into account in calculations of PCSA as it was sufficiently small to be negligible. The physiological cross-sectional areas were converted to muscle forces by multiplying by a muscle stress value of 0.3 Nmm^−2^ [46], [47]. The muscle forces applied to each model are listed in Table 2. Each estimated muscle force was distributed across a number of nodes (between 8 and 30) evenly spread over the corresponding origin site on the skull. Muscle orientations were determined by creating a vector between the origin and the corresponding insertion on a temporary reconstruction of the mandible, which was deleted before solving the FE model. For fan-shaped muscles, such as the temporalis, in which the fascicles radiate from the insertion, varying greatly in their orientation, individual vectors were created for each origin node. The muscle orientations were slightly adjusted between incisor gnawing and molar chewing to account for the propalineal movement of the lower jaw that occurs in the transition between these two feeding modes.

In order to assess the effect of the relative proportions of the masticatory muscles on stress and strain across the skull, further analyses were conducted in which the muscle forces were adjusted on each model to resemble those of the other two rodents. Firstly, the percentage of the total force contributed by each muscle was calculated (Table 2). These percentages were then applied to the total muscle force in each model, in order to redistribute the forces and to put, for example, the rat muscles on the squirrel skull. In order to account for muscles that are not present in all three rodents, the anterior deep masseter and infraorbital part of the zygomatico-mandibularis were assumed to have similar origins and lines of action (Figure 1), and thus to be largely interchangeable. For instance, when applying guinea pig muscle force proportions to the squirrel skull, the percentage of total muscle force found in the guinea pig infraorbital part of the zygomatico-mandibularis was applied to the squirrel anterior deep masseter. The guinea pig deep masseter was assumed to be equivalent to the posterior deep masseter of the rat and squirrel. All possible combinations of skulls and muscles were created (see Table 4 for the muscle forces calculated for each model).

**Table 4:** Recalculated muscle forces (N) simulating muscle proportions of each rodent on the other two skulls.

Skull	Squirrel		Guinea pig		Rat	
Muscle proportions	Guinea pig	Rat	Squirrel	Rat	Squirrel	Guinea pig
Superficial masseter	15.67	7.00	8.23	5.59	8.75	13.33
Anterior deep masseter	4.66	9.35	7.59	10.80	7.47	-
Posterior deep masseter	7.06	13.51			8.07	6.00
Anterior ZM	6.90	1.36	5.50	1.09	5.85	5.87
Posterior ZM	2.04	1.21	1.61	0.97	1.72	1.73
Infraorbital ZM	-	-	7.02	7.48	-	3.97
Temporalis	5.34	11.24	3.35	8.98	3.56	4.54
Internal pterygoid	10.28	8.75	8.20	7.00	8.73	8.74
External pterygoid	3.25	2.77	2.63	2.21	2.79	2.76
**Total**	**55.20**	**55.20**	**44.13**	**44.13**	**46.94**	**46.94**

Abbreviations: ZM, zygomatico-mandibularis.

### Model solution and analysis

The rodent finite element models were solved using Abaqus 6.10.2 (Simulia, Providence, RI, USA). Von Mises stresses for each element were extracted from Abaqus, analysed using R 2.13.1 statistical software (www.r-project.org), and plotted as contour maps of stress and strain across the skulls. Using an especially written script in the Abaqus Python scripting interface, the results from a number of analyses representing bites on all possible teeth were combined, and contour maps were plotted of the maximum stress experienced by each element across all models. The mechanical efficiency of biting in each model was assessed by calculating the ratio of predicted bite force to the applied muscle force [32]. This measure provides an estimate of the efficiency with which muscle force is translated into bite force. To analyse the results of varying muscle configuration on the models, the Abaqus Python scripting interface was used to compute differences between analyses which were then plotted back on to the models as contour maps [48].

It should be noted that the three species under study are different in size, with the rat being the smallest and the guinea pig the largest. The individuals selected for FEA had skull lengths of 43.4 mm (rat), 48.2 mm (squirrel) and 57.5 mm (guinea pig). In contrast to some recent FEA studies, it was decided not to scale the models, either to a uniform surface area [19], [49] or to an allometric scale [50]. Scaling was not felt to be necessary, partly because the differences in size were not great, but mainly because the questions being asked did not require it. By using size-independent variables such as biting efficiency (the ratio of estimated bite force to applied muscle force), and by comparing different muscle configurations on the same skull geometry, the confounding effects of size were avoided.

### Validation

Results of the FEA were validated from *in vivo* measurements of incisor bite forces were obtained from two adult rats (321±8.5 g) and two adult guinea pigs (355±22.6 g) using a Kistler (type 9203) bite force transducer attached to a Kistler charge amplifier (type 5995) and mounted in a custom-built set-up [51]. Ethical approval for AH to conduct the bite force testing was provided by the University of Antwerp ethics committee. Measurements were repeated ten times for each individual and the maximal bite force was retained for comparison with the values predicted by the FE models. Due to the distal position of the teeth and the substantial cheek musculature, it was not possible to gather force data for molar bites. The predicted incisor bite forces were also compared to the limited amount of data available in the published literature [34]. Published data on molar biting is lacking, presumably due to the same practical problems encountered in this study.

## References

[1] Nowak R (1999). Walker's Mammals of the World.

[2] Meng J, Wyss AR, Rose KD, Archibald JD (2005). Glires (Lagomorpha, Rodentia).. The Rise of the Placental Mammals.

[3] Becht G (1953). Comparative biologic-anatomical researches on mastication in some mammals.. Proc Kon Ned Akad Wet, Ser C.

[4] Hiiemae K, Ardran GM (1968). A cinefluorographic study of mandibular movement during feeding in the rat (*Rattus norvegicus*).. J Zool.

[5] Turnbull WD (1970). Mammalian masticatory apparatus.. Fieldiana (Geol).

[6] Wood AE (1965). Grades and clades among rodents.. Evol.

[7] Cox PG, Jeffery N (2011). Reviewing the jaw-closing musculature in squirrels, rats and guinea pigs with contrast-enhanced microCT.. Anat Rec Part A.

[8] Druzinsky RE, Doherty AH, De Vree FL (2011). Mammalian masticatory muscles: homology, nomenclature and diversification.. Integr Comp Biol.

[9] Meng J, Hu Y-M, Li C-K (2003). The osteology of *Rhombomylus* (Mammalia, Glires): Implications for phylogeny and evolution of Glires.. Bull Am Mus Nat Hist.

[10] Druzinsky RE (2010). Functional anatomy of incisal biting in *Aplodontia rufa* and sciuromorph rodents - Part 1: Masticatory muscles, skull shape and digging.. Cells Tissues Organs.

[11] Brandt JF (1855). Untersuchungen über die craniologischen Entwicklungsstufen und Classification der Nager der Jetzwelt.. Mém Acad Imp Sci St Pétersbourg, Sér 6.

[12] Miller GS, Gidley JW (1918). Synopsis of the supergeneric groups of rodents.. J Washington Acad Sci.

[13] Simpson GG (1945). The principles of classification and a classification of mammals.. Bull Am Mus Nat Hist.

[14] Huchon D, Madsen O, Sibbald MJJB, Ament K, Stanhope MJ (2002). Rodent phylogeny and a timescale for the evolution of Glires: Evidence from an extensive taxon sampling using three nuclear genes.. Mol Biol Evol.

[15] Adkins RM, Walton AH, Honeycutt RL (2003). Higher-level systematics of rodents and divergence time estimates based on two congruent nuclear genes.. Mol Phylogenet Evol.

[16] Blanga-Kanfi S, Miranda H, Penn O, Pupko T, Debry RW (2009). Rodent phylogeny revised: analysis of six nuclear genes from all major rodent clades.. BMC Evol Biol.

[17] Weijs WA, Dantuma R (1975). Electromyography and mechanics of mastication in the albino rat.. J Morphol.

[18] Gorniak GC (1977). Feeding in golden hamsters, *Mesocricetus auratus*.. J Morphol.

[19] Byrd KE (1981). Mandibular movement and muscle activity during mastication in the guinea pig (*Cavia porcellus*).. J Morphol.

[20] Hiiemae K (1971). The structure and function of the jaw muscles in the rat (*Rattus norvegicus* L.). III. The mechanics of the muscles.. Zool J Linn Soc.

[21] Satoh K (1997). Comparative functional morphology of mandibular forward movement during mastication of two murid rodents *Apodemus speciosus* (Murinae) and *Clethrionomys rufocanus* (Arvicolinae).. J Morphol.

[22] Satoh K (1998). Balancing function of the masticatory muscles during biting of two murid rodents *Apodemus speciosus* and *Clethrionomys rufocanus*.. J Morphol.

[23] Satoh K (1999). Mechanical advantage of area of origin for the external pterygoid in two murid rodents *Apodemus speciosus* and *Clethrionomys rufocanus*.. J Morphol.

[24] Satoh K, Iwaku F (2006). Jaw muscle functional anatomy in northern grasshopper mouse, *Onychomys leucogaster*, a carnivorous murid.. J Morphol.

[25] Satoh K, Iwaku F (2008). Masticatory muscle architecture in a murine murid, *Rattus rattus*, and its functional significance.. Mammal Study.

[26] Satoh K, Iwaku F (2009). Structure and direction of jaw adductor muscles as herbivorous adaptations in *Neotoma Mexicana* (Muridae, Rodentia).. Zoomorphol.

[27] Druzinsky RE (2010). Functional anatomy of incisal biting in *Aplodontia rufa* and sciuromorph rodents - Part 2: Sciuromorphy is efficacious for production of force at the incisors.. Cells Tissues Organs.

[28] Williams SH, Peiffer E, Ford S (2009). Gape and bite force in the rodents *Onychomys leucogaster* and *Peromyscus maniculatus*: Does jaw-muscle anatomy predict performance?. J Morphol.

[29] Rayfield EJ (2007). Finite element analysis and understanding the biomechanics and evolution of living and fossil organisms.. Ann Rev Earth Planet Sci.

[30] Rayfield EJ (2005). Aspects of comparative cranial mechanics in the theropod dinosaurs *Coelophysis*, *Allosaurus* and *Tyrannosaurus*.. Zool J Linn Soc.

[31] Kupczik K, Dobson CA, Fagan MJ, Crompton RH, Oxnard CE (2007). Assessing mechanical function of the zygomatic region in macaques: validation and sensitivity testing of finite element models.. J Anat.

[32] Dumont ER, Davis JL, Grosse IR, Burrow AM (2010). Finite element analysis of performance in the skulls of marmosets and tamarins.. J Anat.

[33] Dumont ER, Piccirillo J, Grosse IR (2005). Finite-element analysis of biting behavior and bone stress in the facial skeletons of bats.. Anat Rec Part A.

[34] Robins MW (1977). Biting loads generated by the laboratory rat.. Arch Oral Biol.

[35] Bright JA, Rayfield EJ (2011). Sensitivity and *ex vivo* validation of finite element models of the domestic pig cranium.. J Anat.

[36] Curtis N, Witzel U, Fitton L, O'Higgins P, Fagan M (2011). The mechanical significance of the temporal fasciae in *Macaca fascicularis*: an investigation using finite element analysis.. Anat Rec Part A.

[37] Macdonald D (2001). The New Encyclopedia of Mammals.

[38] Wilson DE, Reeder DM (2005). Mammal Species of the World.

[39] Eng CM, Ward SR, Vinyard CJ, Taylor AB (2009). The morphology of the masticatory apparatus facilitates muscle force production at wide jaw gapes in tree-gouging common marmosets (*Callithrix jacchus*).. J Exp Biol.

[40] Cox PG, Fagan MJ, Rayfield EJ, Jeffery N (2011). Biomechanical performance of the rodent skull: sensitivity analyses of finite element models.. J Anat.

[41] Christiansen P (2011). A dynamic model for the evolution of sabrecat predatory bite mechanics.. Zool J Linn Soc.

[42] Bright JA, Rayfield EJ (2011). The response of cranial biomechanical finite element models to variations in mesh density.. Anat Rec Part A.

[43] Chamoli U, Wroe S (2011). Allometry in the distribution of material properties and geometry of the felid skull: Why larger species may need to change and how they may achieve it.. J Theor Biol.

[44] Williams KR, Edmundson JT (1984). Orthodontic tooth movement analysed by the finite element method.. Biomaterials.

[45] Jeffery NS, Stephenson R, Gallagher JA, Jarvis JC, Cox PG (2011). Micro-computed tomography with iodine staining reveals the arrangement of muscle fibres.. J Biomech.

[46] van Spronsen PH, Weijs WA, Valk J, Prahl-Andersen B, van Ginkel FC (1989). Comparison of jaw-muscle bite-force cross-sections obtained by means of magnetic resonance imaging and high resolution CT scanning.. J Dent Res.

[47] Strait DS, Wang Q, Dechow PC, Ross CF, Richmond BG (2005). Modeling elastic properties in finite element analysis: how much precision is needed to produce an accurate model?. Anat Rec Part A.

[48] Pataky TC (2010). Generalized *n*-dimensional biomechanical field analysis using statistical parametric mapping.. J Biomech.

[49] Dumont ER, Grosse IR, Slater GJ (2009). Requirements for comparing the performance of finite element models of biological structures.. J Theor Biol.

[50] McHenry CR, Wroe S, Clausen PD, Moreno K, Cunningham E (2007). Supermodeled sabercat, predatory behaviour in *Smilodon fatalis* revealed by high-resolution 3D computer simulation.. Proc Nat Acad Sci U S A.

[51] Herrel A, Sptihoven L, van Damme R, de Vree F (1999). Sexual dimorphism of head size in *Gallotia galloti*: testing the niche divergence hypothesis by functional analysis.. Func Ecol.

